# Malposition of hemodialysis catheter into the hepatic veins: A case report

**DOI:** 10.12669/pjms.35.2.416

**Published:** 2019

**Authors:** Sibel Kocak Yucel, Ceren Gumusel

**Affiliations:** 1 *Dr. Sibel Kocak Yucel, Department of Nephrology, University of Health Science Bakirkoy, Dr. Sadi Konuk Educational and Research Hospital, Istanbul, Turkey*; 2 *Ceren Gumusel Department of Internal Medicine, University of Health Science Bakirkoy, Dr. Sadi Konuk Educational and Research Hospital, Istanbul, Turkey*

**Keywords:** Malposition, Dialysis catheter, Hepatic vein

## Abstract

Permanent central vein catheter for hemodialysis is a choice for hemodialysis vascular access. Permanent dialysis catheters may be inserted through the jugular, subclavian and femoral veins. It may be inserted when the patient have short life expectancy or not suitable for fistula operation. There may be so many complications for example malposition, hemorrhage and pneumothorax while inserting central venous catheter. Here we present a 44 year old female hemodialysis patient with a malpositioned permanent hemodialysis catheter, catheter tip was found in hepatic vein after three months of insertion.

## INTRODUCTION

In end-stage renal disease (ESRD), renal replacement therapy is achieved by dialysis or renal transplantation. The patients who have ESRD need vascular access for hemodialysis. The vascular access is provided by arterio-venous shunts or dialysis catheters. Catheters may be permanent and temporary. Permanent catheters could be inserted to jugular, subclavian or femoral veins.^1^ Malposition of a central venous catheter is a rare complication (4%).^2^ In this case report, we present ESRD patient who attended our clinic with abdominal pain and dyspnea. Her permanent catheter had been inserted in another medical center and we investigated that the catheter was extended towards the middle hepatic vein using computed tomography (CT).

## CASE REPORT

**Fig.1:**
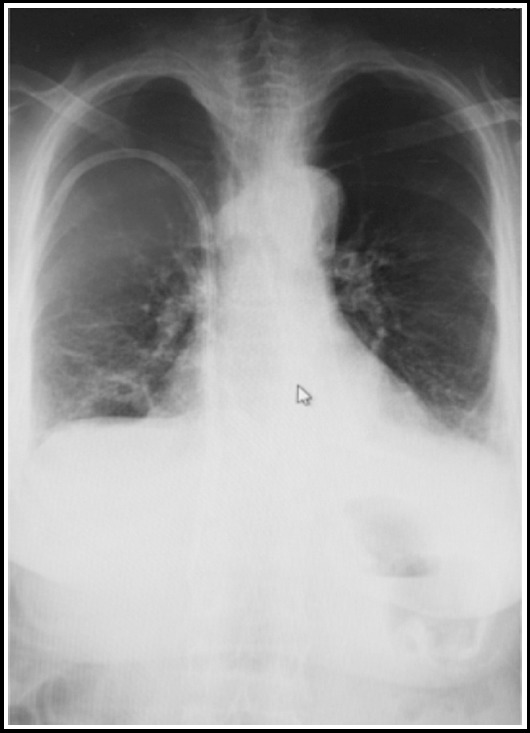
The permanent catheter inserted through the right jugular vein is seen to have extended towards the right upper quadrant.

**Fig.2:**
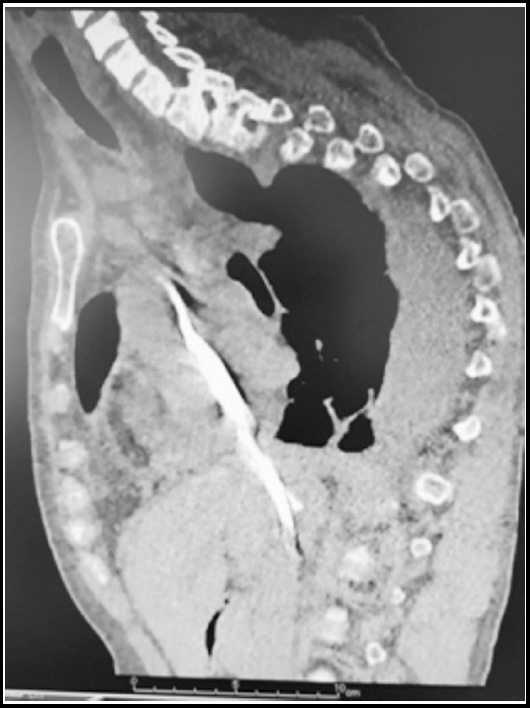
Sagittal oblique reformatted images of the non Contrasted computed tomography scan. The catheter in the jugular vein extends towards the inferior vena cava and into the hepatic vein.

A 44-year-old female patient with ESRD on hemodialysis therapy three times per week for 9 years, was admitted to our hemodialysis center for a regular hemodialysis session program. She had arterial hypertension for 15 years. She had a permanent dual-lumen, cuffed, hemodialysis catheter (diameter 14, 5 Fr, cuff to tip 23 cm) which was inserted through the right jugular vein providing blood flow more than 350 mL/min. She had abdominal pain and dyspnea in dialysis session. Then a chest X-ray (Fig.1) and a thoracic CT (Fig.2) scan were performed and we found that her permanent catheter was inserted through the right jugular vein and had extended through the inferior vena cava and the distal tip of the catheter was ended in the middle hepatic vein. Meanwhile, we learned from the patient history that this catheter was functioning since three months and she was hemodynamically stable during this period. Because of the symptoms, the catheter was immediately removed. After removing the permanent catheter, the symptoms were resolved. Before replacing a new permanent catheter, a venography for upper extremities and superior vena cava was performed by interventional radiology and no flow of contrast agent was observed in superior vena cava vein, suggesting an obstruction in vena cava superior vein and right and left juguler vein’s blood flows were through azygos and hemiazygos veins respectively. So a new functioning permanent, dual-lumen, cuffed, hemodialysis catheter (diameter 14, 5 Fr, cuff to tip 19 cm) was inserted in the right femoral vein.

## DISCUSSION

The use of permanent central vein catheters for hemodialysis is a solution for the access problem of hemodialysis. There are a number of long-term dual-lumen dialysis catheters with or without cuffs. The guidelines about optimal position of central catheters suggest leaving the tip of the catheters in the right atrium. The permanent HD catheter could be inserted into the internal jugular, subclavian, femoral veins and the inferior vena cava. The subclavian vein is not generally recommended for avoiding subclavian vein thrombosis or narrowing. Infectious, mechanical or thrombotic complications of central venous catheter can be observed during insertion of catheter or after then.

Inappropriate catheter placement is the most common reason of below 350 mL/min blood flow velocity. Catheter malposition and subcutaneous flexures may be detected on chest X-rays or by fluoroscopic evaluations and catheter should be repositioned accordingly.^3^ If there is an unexplained chest pain and hypotension right after the dialysis is started, the dialysis must be aborted immediately and the position and functions of the catheter must be checked.^4^ The NKF-DOQI guideline recommends routine chest X-rays to detect potential complications and to verify the placement of the catheter after jugular and subclavian catheter insertion.^5^ The distal tip of the malpositioned catheter may be localized in an improper vein (contralateral subclavian, contralateral brachiocephalic or internal mammarian vein).^6^ Internal thoracic vein, the internal mammarian vein and the pulmonary arteries are the most reported locations for malpositioned central venous catheters. In our case, we discussed a malpositioned functioning permanent catheter in the hepatic vein. A catheter malposition may end up in the hepatic vein as was the case in our study. Four similar cases were found in literature.^7^-^9^ Catheter malposition may lead to fatal outcomes and ineffective dialysis. The malpositioned permanent catheters were diagnosed after a few months from placement when searching for the etiology of nonfunctioning catheter. It is important to check with chest X-rays catheter’s position after insertion.

## CONCLUSION

Central venous catheters are commonly used in end stage renal disease for hemodialysis access. As in cases of the hemodialysis catheter insertion into the right atrium, its tip can be moved or erroneously inserted through the inferior vena cava into the hepatic veins and because of malposition’s of the catheters, clinicians can observe some problems in hemodialysis sessions associated about this issue. Before inserting a catheter risk factors that could cause malposition should be well-identified and after inserting a catheter chest radiograph should be performed to verify the location of the catheter.

### Authors Contribution

**SKY:** treated and followed the patient as primary consultant and did final approval of manuscript.

**CG:** Did write and edited manuscript.
